# Commentary: What visual illusions tell us about underlying neural mechanisms and observer strategies for tackling the inverse problem of achromatic perception

**DOI:** 10.3389/fnhum.2015.00445

**Published:** 2015-08-05

**Authors:** Alan Gilchrist

**Affiliations:** Department of Psychology, Rutgers University/NewarkNewark, NJ, USA

**Keywords:** lightness, brightness, contrast, illumination, depth perception

Blakeslee and McCourt state repeatedly that three terms, lightness, brightness, and brightness contrast, are frequently conflated and/or misused. However, they are apparently unable to give a single example. Instead, they refer to their own (Blakeslee et al., 2008) experiment in which they showed that in their replication of Gilchrist et al. (1983), when illumination appeared homogeneous, lightness and brightness judgments were identical. There is nothing new here. It is well known. They assert that our subjects, who were instructed to make lightness matches, were instead making brightness matches. Our subjects were asked to make lightness matches, they were instructed accordingly, and there is no reason to think that they instead made brightness matches. In our replication (Jacobsen and Gilchrist, 1988) of an earlier Jameson and Hurvich (1961) experiment we obtained both lightness and brightness matches. These were qualitatively different (horizontal vs. diagonal lines). The lightness instructions in the 1988 and 1983 papers were essentially equivalent.

Blakeslee and McCourt claim that my colleagues and I are not comfortable acknowledging the fact that when illumination is homogeneous, lightness and brightness collapse to the same thing. There is no truth to this claim. On page 205 of my book (Gilchrist, 2006) I have written, “Although brightness models could arguably be ignored in a book on surface lightness, there are several reasons to include them. First, under homogeneous illumination, lightness and brightness can be treated as equivalent.”

Blakeslee and McCourt object to my description (2006, p. 6) of brightness as “the perception of a proximal quality—the raw intensity of some part of the image.” They argue that I am confusing brightness with luminance, and they stress that brightness matches deviate substantially from luminance matches. But in a brightness matching task, subjects are instructed to match the luminance of targets, and they try to match luminance, regardless of accuracy. Likewise we say that lightness is perceived reflectance, even though we know that lightness matches deviate from reflectance matches. Brightness is the perception of a proximal quality, even when inaccurate.

However, the central argument made by Blakeslee and McCourt is that my colleagues and I are ignoring an important distinction between what they call apparent lightness and inferred lightness. They contend that when illumination is homogeneous, lightness is perceived directly whereas when a visible illumination edge is present, lightness is not seen directly but merely inferred.

They claim that both in my experiments on depth and lightness (Gilchrist, 1977, 1980) and in our edge substitution experiments (Gilchrist et al., 1983; Gilchrist, 1988), in those conditions in which the subjects reported black and white targets, those targets did not in fact appear black and white, but instead, the subjects merely inferred that those targets were probably black and white. This claim, which was first made in the 1970's by those defending the simplistic lateral inhibition account of lightness, is nonsense. In all of these experiments, subjects simply reported the lightness values as they appeared. Burkamp (1923) showed that fish correctly respond to the reflectance of a feeding tray, even when an illumination edge is present. Are these fish making an inference? Consider Adelson's well-known checkered shadow illusion. The very different appearance of the two equi-luminant targets lying on opposite sides of the illumination edge cannot be attributed to an inference. It occurs immediately and unavoidably. That is the point of the illusion.

It is not hard to see why Blakeslee and McCourt would make such a claim. We have shown that changing the perceived depth of a target or changing whether an edge is perceived as an illumination edge or as a reflectance edge can move the perceived lightness of a target almost from one end of the lightness scale to the other, with merely a trivial change in the retinal image. Unless those data can be discredited, the ODOG model cannot account for lightness perception in most real-world scenes.

## Conflict of interest statement

The author declares that the research was conducted in the absence of any commercial or financial relationships that could be construed as a potential conflict of interest.
